# Electrophysiological and Morphological Characterization of Chrna2 Cells in the Subiculum and CA1 of the Hippocampus: An Optogenetic Investigation

**DOI:** 10.3389/fncel.2018.00032

**Published:** 2018-02-13

**Authors:** Heather Nichol, Bénédicte Amilhon, Frédéric Manseau, Saishree Badrinarayanan, Sylvain Williams

**Affiliations:** ^1^Department of Psychiatry, Douglas Mental Health University Institute, McGill University, Montreal, QC, Canada; ^2^Department of Neuroscience, CHU Sainte-Justine Research Center, Université de Montréal, Montreal, QC, Canada

**Keywords:** oriens lacunosum-moleculare, interneuron, GABA, GABA_B_ receptors, subiculum, hippocampus

## Abstract

The nicotinic acetylcholine receptor alpha2 subunit (Chrna2) is a specific marker for oriens lacunosum-moleculare (OLM) interneurons in the dorsal CA1 region of the hippocampus. It was recently shown using a Chrna2-cre mice line that OLM interneurons can modulate entorhinal cortex and CA3 inputs and may therefore have an important role in gating, encoding, and recall of memory. In this study, we have used a combination of electrophysiology and optogenetics using Chrna2-cre mice to determine the role of Chrna2 interneurons in the subiculum area, the main output region of the hippocampus. We aimed to assess the similarities between Chrna2 subiculum and CA1 neurons in terms of the expression of interneuron markers, their membrane properties, and their inhibitory input to pyramidal neurons. We found that subiculum and CA1 dorsal Chrna2 cells similarly expressed the marker somatostatin and had comparable membrane and firing properties. The somas of Chrna2 cells in both regions were found in the deepest layer with axons projecting superficially. However, subiculum Chrna2 cells displayed more extensive projections with dendrites which occupied a significantly larger area than in CA1. The post-synaptic responses elicited by Chrna2 cells in pyramidal cells of both regions revealed comparable inhibitory responses elicited by GABA_A_ receptors and, interestingly, GABA_B_ receptor mediated components. This study provides the first in-depth characterization of Chrna2 cells in the subiculum, and suggests that subiculum and CA1 Chrna2 cells are generally similar and may play comparable roles in both sub-regions.

## Introduction

The hippocampus is a multi-modal structure long known to serve critical functions in spatial information processing, learning, and memory. Interneurons are believed to play a central role in hippocampal function, being at the interface between the hippocampus network which they can powerfully modulate, and inputs to this network (Freund and Buzsáki, 1996; McBain and Fisahn, 2001; Klausberger and Somogyi, 2008). Among the diverse hippocampal interneuron populations, the oriens lacunosum-moleculare (OLM) interneuron is poised to make a significant contribution to the modulation of hippocampal inputs and the regulation of hippocampal activity. These CA1 GABAergic, somatostatin (Som)-expressing interneurons are named for their distinctive morphology: their soma and dendritic trees are located in the stratum oriens and their axons extend directly out to arborize in the stratum lacunosum-moleculare (SLM; Cajal, 1911; McBain et al., 1994; Sik et al., 1995; Maccaferri et al., 2000; Losonczy et al., 2002; Leão et al., 2012). Recently, the nicotinic acetylcholine receptor Chrna2 was identified as a specific genetic marker for CA1 OLM interneurons (Leão et al., 2012). Chrna2 was also found to be expressed in Som-expressing cells of the subiculum (Leão et al., 2012); however, the electrophysiological and morphological properties of these cells have not yet been characterized. Though the subiculum is a largely under-investigated hippocampal sub-region, an enhanced understanding of the subiculum is critical as it is poised to make a significant contribution to hippocampal function.

The subiculum is the major output structure of the hippocampus (Swanson and Cowan, 1977; Köhler, 1985, 1990; Witter et al., 1990; Canteras and Swanson, 1992; O’Mara et al., 2001). It has been linked to a number of functions including learning and memory (Galani et al., 1997; Laxmi et al., 1999; Oswald and Good, 2000). Given that Chrna2-expressing OLM interneurons in CA1 are postulated to play a role in memory formation through the attenuation of entorhinal cortex (EC) input (Lovett-Barron et al., 2014), a better understand of subiculum Chrna2 cells and their connectivity to pyramidal cells receiving EC input may provide insight into their role in subiculum function and in learning and memory.

The subiculum has also been found to acts as an intrinsic generator of hippocampal theta rhythm (Jackson et al., 2014), oscillations of 3–12 Hz believed to play an important role in hippocampal function (Buzsáki, 2002; Colgin, 2013; Huh et al., 2016). OLM interneurons in CA1 have also been associated with theta rhythm (Huh et al., 2016). They are known to fire spontaneously at theta frequency, increase their firing during theta rhythm and phase lock to the trough of the theta cycle in stratum pyramidale (Maccaferri and McBain, 1996; Pike et al., 2000; Gillies et al., 2002; Klausberger et al., 2003; Gloveli et al., 2005; Varga et al., 2012; Katona et al., 2014). Accordingly, they may phase-modulate the EC input to the distal dendrites of pyramidal cells, inputs which contribute to the generation of theta rhythm (Kamondi et al., 1998; Buzsáki, 2002; Mizuseki et al., 2009). A better understanding of Chrna2 cells in the subiculum would provide insight into whether these cells contribute to theta rhythm like their counterparts in CA1.

The goal of this study is to characterize Chrna2 cells in the subiculum compare them to Chrna2 cells in CA1. Using whole-cell patch clamp and optogenetic techniques, we characterized subiculum Chrna2 cells and the post-synaptic responses they elicit in pyramidal cells. These properties were compared to those of CA1 Chrna2 cells to determine whether subiculum Chrna2 cells are a distinct population. We found that subiculum and CA1 cells were similar, with respect to their Som expression and electrophysiological properties. While subiculum Chrna2 cells displayed more extensive dendritic trees and deep layer axonal branching than those in CA1, they evoked similar post-synaptic inhibitory responses in pyramidal cells.

## Materials and Methods

### Animals

This study was carried out in accordance with the recommendations of the Canadian Council on Animal Care and the McGill University Animal Care Committee. The protocol was approved by the McGill University Animal Care Committee. Animals were housed in a temperature-controlled room with a 12/12 h light/dark cycle and food and water *ad libitum*.

This study made use of *Chrna2-cre* transgenic C57BL/6 mice (Chrna2 mice, Lab of Richardson Leão, Uppsala, Sweden). In this recently developed mouse line, cre recombinase is expressed under the Chrna2 promoter (Leão et al., 2012). As described above, Chrna2 is expressed exclusively in OLM interneurons in dorsal CA1 (Leão et al., 2012); thus, this mouse line allows for the specific identification and targeting of this distinct interneuron population. Chrna2 mice were also crossed with homozygote Ai9 lox-stop-lox-tdTomato cre-reporter strain mice (RRID:IMSR_JAX:007905) to generate Chrna2-Tom mice in which tdTomato (Tom) is exclusively expressed in cells expressing cre recombinase. Tom fluorescence was used to identify Chrna2 cells.

### Quantification of Somatostatin Expression

Immunohistochemistry experiments were used to determine whether Chrna2 cells in the subiculum express Som. Chrna2-Tom animals were intracardially perfused with paraformaldehyde (PFA) and brains were harvested as described in Amilhon et al. (2015). Brains were sectioned into 25 μm thick coronal slices using a cryostat (Leica CM3050S, Germany). Slices (one slice per 100 μm for the full rostrocaudal extent of the hippocampus) were incubated with a rabbit anti-Som primary antibody (1:250, Santa Cruz Biotechnology, Cat# sc-13099, RRID:AB_2195930) for 16 h at 4°C, followed by an anti-rabbit secondary antibody coupled to Alexa488 (1:1000, Molecular Probes, Cat# A-21206, RRID:AB_141708) for 2 h at room temperature to visualize Som+ cells. Slices were mounted and one image was acquired at 10× magnification in both CA1 and subiculum for each slice using an Axio Observer microscope (Carl Zeiss, Germany). Som+ and Tom+ cells were counted using ImageJ software^1^ (RRID:SCR_003070).

### Electrophysiological Properties of Chrna2 Cells

To assess the electrophysiological properties of CA1 and subiculum Chrna2 cells, patch clamp experiments were performed in Chrna2-Tom animals. Mice were killed by decapitation, and the brain was dissected and placed in an ice-cold high-sucrose solution (252 mM sucrose, 24 mM NaHCO_3_, 10 mM glucose, 3 mM KCl, 2 mM MgCl_2_, 1.25 mM NaH_2_PO_4_, and 1 mM CaCl_2_, continuously oxygenated with 95% O_2_/5% CO_2_; pH 7.3). Horizontal or coronal slices (300 or 400 μm) were cut using a vibratome (Leica VT1000S, Germany) and transferred to an artificial cerebrospinal fluid (aCSF) solution at room temperature (solution as above with 126 mM NaCl replacing sucrose, 4.5 mM KCl and 2 mM CaCl_2_) for 30 min before recording. Slices were recorded in a bath of aCSF perfused at a rate of 2 ml/min and heated to 30°C (Temperature controller TC-324B, Warner Instruments, Hamden, CT, United States). Chrna2 cells were identified by Tom fluorescence using an upright BX51WI Olympus microscope with a 60× immersion objective (Olympus Canada, Richmond Hill, ON, Canada) and an X-cite Series 120Q fluorescence system (Lumen Dynamics, Mississauga, ON, Canada). Glass patch pipettes (Warner Instruments, Hamden, CT, United States) had a resistance 2.0–6.0 MΩ and were filled with intra-pipette solution (144 mM K-gluconate, 10 mM HEPES, 3 mM MgCl_2_, 2 mM Na_2_ATP, 0.3 mM GTP, and 0.2 mM EGTA; adjusted to pH 7.2 with KOH). Chrna2 cells were recorded using a visually guided whole-cell patch clamp technique, a MultiClamp 700B amplifier, a DigiData 1440A digitizer and pClamp10 software, and analyzed with Clampfit10 Software (for all: Molecular Devices, Sunnyvale, CA, United States). Recordings were kept for analysis only if spikes overshot 0 mV and access resistance was <30 MΩ. There was no correction for junction potential (-9 mV). Membrane resistance (R_m_) and access resistance (R_a_) were measured in voltage clamp (vc) using pClamp10 software. The following properties were assessed in current clamp (cc). Resting membrane potential (*V*_r_) and spontaneous spiking were assessed over a 30 s recording with no holding current. To assess spike properties, cells were held at a holding potential (h.p.) of -60 mV and a series of 600 ms depolarizing current steps was applied. The step which elicited the first spike was used to assess spike amplitude and half width, and after-hyperpolarization (AHP) amplitude and time. The same series of current steps was applied at an h.p. of -80 mV. The current step which elicited the first spike at -80 mV was noted and the response to a step of equal current applied at -60 mV was analyzed to determine firing rate, peak interspike interval (ISI) (ISI for the first two spikes), steady state ISI (ISI for the last two spikes), and accommodation [(steady state ISI - peak ISI)/steady state ISI]. To assess sag, if present, a series of hyperpolarizing current steps was applied at an h.p. of -60 mV. The step which hyperpolarized the cell to -120 mV was used to calculate sag amplitude, measured as the difference between peak and steady state hyperpolarization, and to determine the presence of a rebound spike. The properties of subiculum and CA1 cells were compared.

### Morphology of Chrna2 Cells

To characterize the morphology of Chrna2 cells in CA1 and subiculum, cells were patched using the above methodology with the addition of biocytin (0.4%, UNC Joint Vector Laboratories, RRID:SCR_002448) to the intra-pipette solution. To obtain efficient biocytin fills, cells were subjected to a high amount of current (700–800 pA for 100 ms) at the end of each recording session (Jiang et al., 2015). Following this, the pipette was left undisturbed in whole-cell mode for 30 min to allow for the diffusion of biocytin through all neuronal processes. Finally, the pipette was retracted carefully to ensure that the cell membrane did not rupture, as this would result in incomplete fills or the loss of the soma.

Slices were fixed in PFA (4% in PBS) at 4°C overnight. Fixed slices were incubated with streptavidin Alexa 488 (1:1000, Jackson ImmunoResearch, West Grove, PA, United States) in 0.3% PBS-Triton overnight at room temperature. Slices were mounted on glass slides with Fluoromount G (Southern Biotech, Canada, 0100-01).

Biocytin-labeled Chrna2 cells were imaged under the MBF Zeiss ApoTome.2 microscope with a 20× magnification (Carl Zeiss, Germany). The cells were reconstructed manually using Neurolucida 11 software (MBF Bioscience, Williston, VT, United States). Dendrites were distinguished from axons by the presence of spines. Initial axon segments were identified by their smooth appearance near the soma which later appeared discontinuous as the axon continued branching. Due to slicing techniques, neuronal processes may be cleaved, leaving abrupt endings visualized as ball-like structures at the end of the processes. The dendrites and axons for cells were noted for abrupt endings, and all cells that were filled for 30 min and had no visualized swellings in their axonal or dendritic processes were considered for neuron tracing. Area and perimeter occupied by the dendritic tree were assessed using convex hull analysis. Dendritic complexity was assessed using Sholl analysis (Sholl, 1953), in which concentric Sholl segments are generated starting from the center of the cell body (radial interval: 10 μm) and the number of process intersections per Sholl segment are recorded. Both analyses were conducted with Neurolucida 11 software (MBF Bioscience, Williston, VT, United States).

### Optogenetic Activation of Chrna2 Cells

To investigate post-synaptic responses elicited by Chrna2 cell activation in pyramidal cells, an optogenetics approach was employed. This allowed the precise activation of Chrna2 cells. The opsin ChETA, a humanized variant of the excitatory opsin ChR2 [hChR2(E123T/T159C)] with faster on/off kinetics and improved spike fidelity at higher frequency stimulation (Berndt et al., 2011) was delivered using a cre-dependent adeno-associated viral vector (AAVdj). To deliver the opsin, post-natal day 15–16 Chrna2 or Chrna2-Tom mice were anesthetized with isoflurane and placed in a stereotaxic frame (Stoelting, Wood Dale, IL, United States). AAVdj-EF1α-DIO-ChETA-eYFP (OHSU Molecular Virology Support Core, RRID:SCR_012730) was injected at the dorsal CA1/subiculum border [0.6 μl at rate of 0.06 μl/min, needle left in place for additional 5 min; coordinates (from bregma): lateral, ±3.00; anteroposterior, -2.70; dorsoventral, -2.05]. Following surgery, animals were returned to their home cage. Experiments were performed 2–4 weeks post-surgery to allow for virus expression.

At post-injection day 22–24, Chrna2-Tom animals were perfused (protocol as described above) for use in immunohistochemistry experiments to validate virus expression. The immunohistochemistry protocol was as described above with the following antibodies: a goat anti-GFP primary antibody, reactive with eYFP (1:5000, Novus, Cat# NB 100-1770, RRID:AB_523903), and a rabbit anti-RFP primary antibody, reactive with Tom (1:10,000, Rockland, Cat# 600-401-379, RRID:AB_2209751), followed by an anti-goat secondary antibody coupled to Alexa488 (1:1000, Molecular Probes, Cat# A-11055, RRID:AB_142672) and an anti-rabbit secondary antibody coupled to Alexa568 (1:1000, Molecular Probes, Cat# A-11036, RRID:AB_143011). Slices were imaged using a Zeiss Imager.M2 microscope with a 10× objective (Carl Zeiss, Germany) and Stereo Investigator 13 software (RRID:SCR_002526). The injection site was located, and one slice per 100 μm was analyzed for 500 μm rostral and caudal to this location. One image stack was acquired at 10× magnification in both CA1 and subiculum for each slice, and YFP+ and Tom+ cells were counted using ImageJ software (see text footnote 1, RRID:SCR_003070).

To characterize the optogenetic activation of Chrna2 cells, animals were used in patch clamp experiments at post-injection day 16–25. (Patch clamp protocol as described above. No neurobiotin in intra-pipette solution for this and all subsequent experiments.) A 1 mm optic fiber connected to a blue LED (474 nm or 447 nm, Luxeon Start LEDs, Brantford, ON, Canada) was positioned over the hippocampus (maximum light power 49 mW for 474 nm or 43 mW for 447 nm, at fiber tip). To first ensure that Chrna2 cells could be driven by blue light, ChETA-expressing Chrna2 cells, as identified by YFP expression, were patched and responses to blue light pulses were recorded. Photocurrent was measured at the end of a 500 ms light pulse in vc at an h.p. of -70 mV (mean of 10 recordings per cell). Spiking parameters were assessed in cc during light pulses of 1, 5, 10, 20, 50, and 500 ms at an h.p. of -60 mV. Frequency of firing was measured from 500 ms light pulses (mean of 10 recordings per cell). The mean delay between light onset and spike start was measured for the first spike elicited by a 500 ms light pulse (mean of three recordings per cell). To assess spike fidelity, light pulses of 1, 5, and 10 ms were delivered in trains of 1, 2, 4, 6, 8, 10, 20, 40, 60, 80, and 100 Hz (mean of 10 recordings per cell per condition, 100 Hz not assessed for 10 ms pulses).

To characterize pyramidal cell post-synaptic responses elicited by Chrna2 cell activation, putative pyramidal cells, as identified by shape, position, and firing properties, were patched in subiculum and CA1 during optogenetic stimulation of Chrna2 cells. Regular vs burst firing subiculum pyramidal cells were identified by responses to 600 ms depolarizing current steps. Burst firing pyramidal cells displayed at least one burst during the current step (burst = two or more spikes at a frequency ≥100 Hz). Responses to blue light pulses of 5, 10, 20, and 50 ms were recorded in both vc and cc at h.p.’s from -45 to -80 mV in steps of 5 mV. Analyses were conducted on the mean of 10 recording per condition for each cell. If a spontaneous event, such as a spike, obscured the post-synaptic response, this recording was omitted from the mean. Any condition for which fewer than three recordings remained after these omissions was not included in the analysis. Amplitude, half width, decay time, and delay between light onset and post-synaptic response start or peak were analyzed and compared between pulse widths and pyramidal cell types at an h.p. of -60 mV. This h.p. was chosen as it was the potential closest to mean *V*_r_ (-56.3 ± 1.2 mV) that was far enough from spike threshold to allow little to no spiking in all cells. Amplitude of the response was examined in vc across different h.p.’s to determine the reversal potential. Paired pulse recordings were also performed in vc for 10 ms light pulses at an h.p. of -60 mV at delays of 50, 100, 200, 500, and 1000 ms. Paired pulse ratio was calculated as amplitude of second/first response from the mean of 10 recordings per condition.

To determine which receptors play a role in post-synaptic responses to optogenetic activation of Chrna2 cells, pharmacological methods were employed to block GABA receptors. GABA_A_Rs were blocked using SR95531 (gabazine, 5 μM, Abcam, United Kingdom). GABA_B_Rs were blocked using CGP-55845 hydrochloride (CGP-55845, 1 μM, Abcam, United Kingdom). Responses to blue light pulses of 5, 10, 20, and 50 ms were recorded in cc at h.p.’s from -45 to -80 mV (protocols and analysis as described above) after the addition of gabazine, and again after the subsequent addition of CGP-55845. Amplitude, half width, decay time, and delay between light onset and post-synaptic response start or peak were analyzed at an h.p. of -60 mV and pulse width of 50 ms. This pulse width was chosen as, in all but one cell (*n* = 14/15), a component of the response remained after gabazine application; a larger proportion than for other pulse widths.

### Statistical Analysis

All data were tested for normality using the Shapiro–Wilk test. Normally distributed data were compared with the following parametric tests: two-tailed one-sample Student’s *t*-test for paired-pulse ratios, two-tailed paired or unpaired Student’s *t*-tests for two-group comparisons and two-way ANOVAs with Bonferroni *post hoc* two-tailed paired Student’s *t*-tests for multi-factor comparisons. ANOVAs were corrected for sphericity with the Greenhouse–Geisser correction. Data that were not normally distributed were compared with the following non-parametric tests: two-tailed Mann–Whitney (unpaired) or Wilcoxon (paired) tests for two-group comparisons and a combination of Kruskal–Wallis (interactions and cell type main effect) and Friedman tests (pulse width main effect) with Bonferroni corrected Wilcoxon two-tailed *post hoc* tests for multi-factor comparisons. Within group variance was compared across the two regions using Levene’s test. In cases of multiple comparisons, *p*-values for one-sample test, two-group tests or for interaction effects and main effects in multi-factor tests were adjusted using the Bonferroni–Holm correction. Statistical tests were performed using SPSS (SPSS version 22, RRID:SCR_002865), VassarStats^2^ (RRID:SCR_010263) or custom statistics programs (created by Dr. Joseph Rochford, McGill University, Montreal, QC, Canada). Means are listed with ±standard error of the mean (SEM).

## Results

To determine whether subiculum Chrna2 cells are a distinct population, Chrna2 cells were characterized according to their Som expression, electrophysiological properties and morphology, and compared to their counterparts in CA1. Post-synaptic responses elicited by Chrna2 cells in subiculum and CA1 pyramidal cells were also characterized and compared. Finally, we aimed to determine whether subiculum pyramidal cells also received EC input, which may be modulated by Chrna2 cells.

### Somatostatin Expression

**FIGURE 1:**
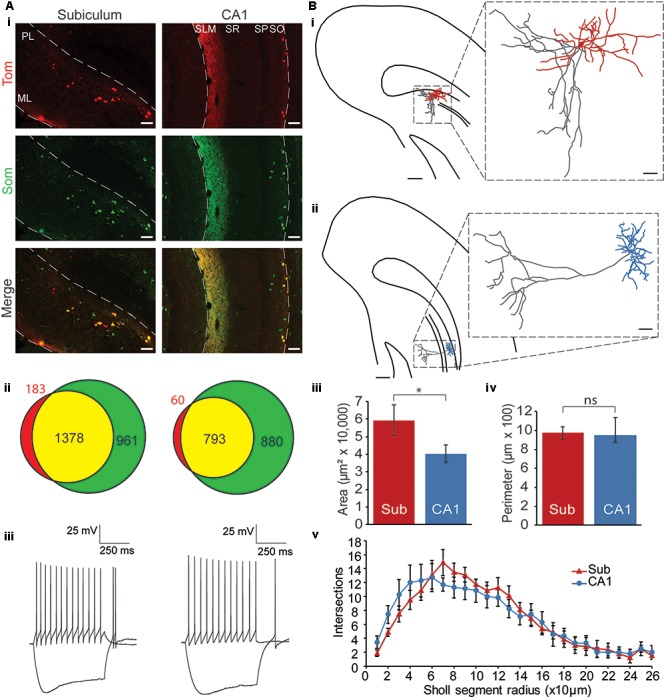
Characterization of Chrna2 cells. **(Ai)** Immunohistochemistry. 10× Images from a Chrna2-Tom animal treated with an anti-Som antibody. Chrna2 cell are visualized with Tom fluorescence in red. Som+ cells are in green. Co-expressing cells are shown in yellow. Left: subiculum. Right: CA1. ML, molecular layer; PL, polymorphic layer; SLM, stratum lacunosum-moleculare; SR, stratum radiatum; SP, stratum pyramidale; SO, stratum oriens. Scale bar = 100 μm. **(Aii)** Venn diagrams depicting proportions of Som+ and Tom+ cells. Tom+Som– cells in red, Tom+Som+ cells in yellow, and Tom–Som+ cells in green. Exact number of cells as indicated. Left: subiculum. Right: CA1. Animals: *n* = 4. Cells: subiculum, *n* = 2522; CA1, *n* = 1733. **(Aiii)** Representative Chrna2 cells recorded in subiculum (left) and CA1 (right). Traces showing current clamp (cc) recordings of responses to depolarizing and hyperpolarizing current steps. **(Bi)** Representative reconstruction of a subiculum Chrna2 cell. Hippocampus borders and pyramidal layer outlined. Soma and dendrites in red. Axon in gray. Scale bar = 250 μm. Inset: Enlarged reconstruction. Scale bar = 50 μm. **(Bii)** As in **(Bi)** for a CA1 Chrna2 cell. Soma and dendrites in blue. Axon in gray. **(Biii)** Mean area and **(Biv)** perimeter of the reconstructed dendritic trees of Chrna2 cells in subiculum (Sub, *n* = 8) and CA1 (*n* = 7). ^∗^*p* < 0.05. ns = not significant, *p* > 0.05. Error bars = SEM. **(Bv)** Number of intersections per Sholl segment for reconstructed dendritic trees of Chrna2 cells in subiculum (red, *n* = 8) and CA1 (blue, *n* = 7).

To address this first objective, immunohistochemistry experiments were performed with Chrna2-Tom mice (*n* = 4; **Figure 1Ai**). Tom+ and Som+ cells were counted in subiculum and CA1 (subiculum, *n* = 2522; CA1, *n* = 1733; **Figure 1Aii**). In subiculum, 88 ± 1% of Tom+ cells were Som+. In CA1, this proportion was significantly higher at 93 ± 1% [two proportion *t*-test with Bonferroni–Holm correction, *t*(2412) = 3.7, *p* < 0.01]. Overall, however, as in CA1, the majority of subiculum Chrna2 cells express Som (**Figure 1Aii**). The proportion of Som+ cells that are Tom+ was also assessed, and found to be significantly greater in subiculum than in CA1 at 59 ± 2 and 47 ± 3%, respectively [two-proportion *t*-test with Bonferroni–Holm correction, *t*(4010) = 7.2, *p* < 0.01]. This suggests that Chrna2 cells represent a greater proportion of the Som+ interneuron population in subiculum than in CA1 (**Figure 1Aii**).

### Electrophysiological Properties

To assess the electrophysiology properties of subiculum Chrna2 cells, patch-clamp experiments were performed in Chrna2-Tom animals. Chrna2 cells were recorded in subiculum and CA1 (*n* = 18 and 16, respectively), and their resting membrane potentials, responses to depolarizing and hyperpolarizing current steps, and spike properties, were assessed (**Figure 1Aiii** and **Table 1**). Chrna2 cells in both subiculum and CA1 fired spontaneously at rest at slow frequency, responded to depolarizing current steps with regular firing, and displayed a sag in their response to hyperpolarizing current steps (**Table 1**). No significant differences were found for any of the electrophysiological properties examined [unpaired two-tailed *t*-tests, *t*(32)’s ≤ 1.7; Mann–Whitney tests, *U*(16,18)’s ≤ 201; and two-proportion *t*-tests, *t*(32)’s ≤ 0.9 with Bonferroni–Holm correction, *p* > 0.05 for all]. Furthermore, there was no significant difference in variance between subiculum and CA1 for any of the electrophysiological properties examined [Levene’s tests with Bonferroni–Holm correction, *W*(32)’s ≤ 4.5, *p* > 0.05 for all].

**Table 1:** Electrophysiological properties of Chrna2 cells recorded in subiculum and CA1.

	Subiculum (*n* = 18)	CA1 (*n* = 16)
R_m_ (MΩ)	387.8 ± 47.2	378.1 ± 35.6
R_a_ (MΩ)	15.7 ± 1.3	14.3 ± 1.1
*V*_r_	-49.9 ± 1.5	-52.3 ± 1.3
Spontaneous spiking	83%	94%
Frequency of spontaneous spiking (Hz)	4.5 ± 1.9	6.4 ± 1.9
Firing rate (Hz)	26 ± 2.1	23.4 ± 2.4
Max firing ISI (ms)	35.8 ± 3.1	45.7 ± 5.6
Steady state ISI (ms)	49.8 ± 3.6	54.3 ± 5.5
Accommodation (%)	26.7 ± 4.2	15.9 ± 4.8
Sag amplitude (mV)	11.5 ± 1.7	11.7 ± 1.7
Rebound spike (%)	61	63
Spike amplitude (mV)	91.8 ± 1.4	91.2 ± 2.5
Spike half width (ms)	1.5 ± 0.1	1.2 ± 0.1
AHP amplitude (mV)	-17.2 ± 0.7	-18.0 ± 0.6
AHP time (ms)	136.5 ± 13.1	145 ± 13.1

±*SEM*.

### Morphology

To assess the morphology of Chrna2 cell in subiculum and compare this morphology to CA1, cells were filled with biocytin in patch clamp experiments and reconstructed (subiculum, *n* = 8; CA1, *n* = 7; **Figures 1Bi–Bv**). In CA1 Chrna2 cells, the soma and horizontally oriented dendrites were located in the stratum oriens (**Figure 1Bii**). The axon extended directly out toward SLM, with few collaterals to stratum oriens. Somas for cells in both regions were found to be uniformly ovoid in structure. Like in CA1, the somas of subiculum Chrna2 cells were located in the deepest layer, the polymorphic layer. However, they appeared to be more widely dispersed than in CA1, a feature apparent when visualizing Tom fluorescence in Chrna2-Tom animals (**Figure 1Ai**). Rather than the narrow band of stratum oriens occupied by Chrna2 cell somas in CA1, the somas of Chrna2 cells in subiculum occupied the full extent of the polymorphic layer, sometimes extending into the deep pyramidal layer. The dendrites of subiculum Chrna2 cells also extended more broadly and gave rise to more extensive dendritic branching than those seen in CA1 (**Figure 1Bi**). Convex hull analysis showed that the dendritic tree of subiculum Chrna2 cells occupied a significantly greater area than in CA1 [**Figure 1Biii**; Mann–Whitney test: *U*(8,7) = 46, *p* = 0.04]. There was no significant difference dendritic tree perimeter (**Figure 1Biv**) [unpaired two-tailed *t*-test: *t*(13) = 0.24, *p* = 0.81]. Sholl analysis showed no significant difference in the number of intersections between Chrna2 cells in subiculum and CA1, but did show a significant effect of radius with proximal and intermediate Sholl segments (20–160 μm) having more intersections than more distal segments (**Figure 1Bv**) [two-way ANOVA with Bonferroni *post hoc* tests for Sholl segment radii which included two or more cells per group (10–260 μm). Interaction: *F*(1,25) = 0.9, *p* = 0.45. Region main effect: *F*(1) = 0.002, *p* = 0.96. Radius main effect: *F*(25) = 31.1, *p* < 0.001 with *post hoc t*-test with Bonferroni correction: *p* < 0.05 for 20–30 μm vs ≥220 μm; 40, 50, and 160 μm vs ≥210 μm; 60 and 150 μm vs ≥190 μm; 140 μm vs ≥180 μm; 70 and 130 μm vs ≥170 μm; and 80–120 μm vs ≥160 μm]. Axon branching was also more extensive and more variable in subiculum Chrna2 cells than in CA1, with axon collaterals projecting within the polymorphic layer in all cells (**Figure 1Bi**). For almost all cells, the axons emerged directly from the soma. Unlike in CA1, only four of eight subiculum Chrna2 cells had axons extending superficially to the molecular layer. Additionally, two of eight cells in the subiculum formed local axonal arbors around the soma. This was absent in the CA1. Overall, the soma and dendrites of subiculum Chrna2 cells occupied a broader area than those in CA1, and the axons of subiculum Chrna2 cells extended to both deep and superficial layers, whereas CA1 Chrna2 cells extended primarily superficially.

### Post-synaptic Responses

**FIGURE 2:**
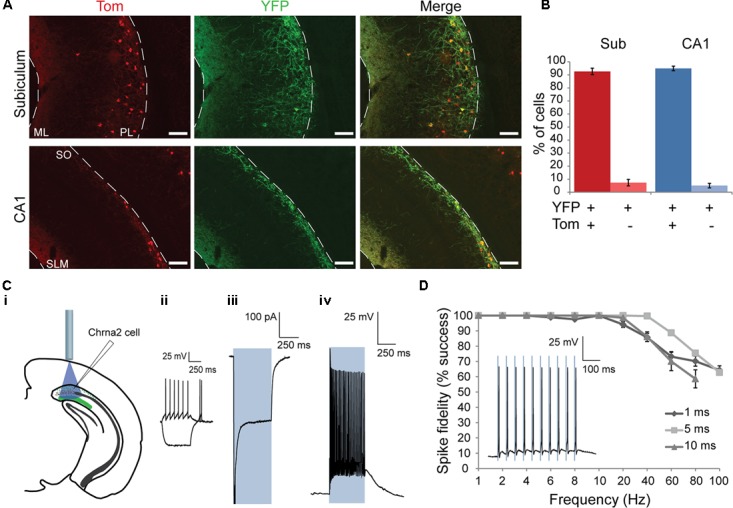
Anatomical and electrophysiological characterization of ChETA-YFP expression in Chrna2 cells. **(A)** 10× Images from a Chrna2-Tom animal injected with AAVdj-DIO-ChETA-eYFP in CA1/subiculum. Chrna2 cells are visualized with Tom fluorescence in red. ChETA-expressing cells are visualized with YFP fluorescence in green. Co-expressing cells are shown in yellow. Top: subiculum. Bottom: CA1. ML, molecular layer; PL, polymorphic layer; SLM, stratum lacunosum-moleculare; SO, stratum oriens. Scale bar = 100 μm. **(B)** Mean proportion of YFP+ cells that were Tom+ or Tom– in subiculum (red) and CA1 (blue). Animal: *n* = 3. Cells: subiculum, *n* = 522; CA1, *n* = 308. **(Ci–Civ)** Patch clamp recordings from a representative subiculum Chrna2 cell expressing ChETA. **(Ci)** Diagram depicting optogenetic patch clamp experiment. **(Cii)** Responses to depolarizing and hyperpolarizing current steps in cc. **(Ciii)** Mean recorded photocurrent produced by a 500 ms light pulse (blue box) in voltage clamp (vc) at a holding potential (h.p.) of –70 mV. Spike peak was truncated to expand view of plateau photocurrent. **(Civ)** Spiking in response to a 500 ms light pulse (blue box) in cc at an h.p. of –60 mV. **(D)** Mean spike fidelity for trains of 1, 5, and 10 ms light pulses at frequencies of 1–100 Hz. *n* = 7–9. Error bars = SEM. Inset: Spiking in response in a representative cell to an 8 Hz train of 5 ms light pulses (blue boxes) in cc at an h.p. of –60 mV.

An optogenetic approach was used to investigate the properties of the post-synaptic responses elicited by Chrna2 cells in CA1 and subiculum pyramidal cells. To activate Chrna2 cells, the blue light sensitive excitatory opsin ChETA was expressed using a cre-dependent viral vector. To first validate opsin expression, the brains of Chrna2-Tom animals injected with AAVdj-EF1α-DIO-ChETA-eYFP were examined using immunohistochemistry (*n* = 3). Tom+ Chrna2 cells and YFP+ ChETA-expressing cells were counted in subiculum and CA1 (subiculum, *n* = 522; CA1, *n* = 308; **Figures 2A,B**). The YFP+ population was assessed to determine the proportion of cells that were either Tom+ or Tom-. The majority of YFP+ cells in both subiculum and CA1 (**Figure 2B**) were also Tom+ (95 ± 2 and 93 ± 2%, respectively). These proportions were not significantly different [two-proportion *t*-test, *t*(828) = 1.9, *p* = 0.06]. This suggests that virus expression was specific to Chrna2 cells in both the subiculum and CA1.

It was next determined how efficiently spiking could be activated by blue light pulses in ChETA-expressing Chrna2 cells. YFP+ cells were recorded and responses quantified (**Figures 2Ci–Civ,D**; *n* = 20: 15 subiculum and 5 CA1) using the same stimulation parameters. Mean photocurrent was found to be -293.5 ± 42.3 pA. The mean minimum pulse width required to elicit a spike was 1.4 ± 0.3 ms and the mean firing frequency over a 500 ms light pulse was 50.4 ± 6.7 Hz. Mean delay between light pulse onset and spike onset was found to be 3.5 ± 0.3 ms. Spike fidelity was robust (mean >90%) for frequencies up to and including 20 Hz (*n* = 7–9) (**Figure 2D**).

**FIGURE 3:**
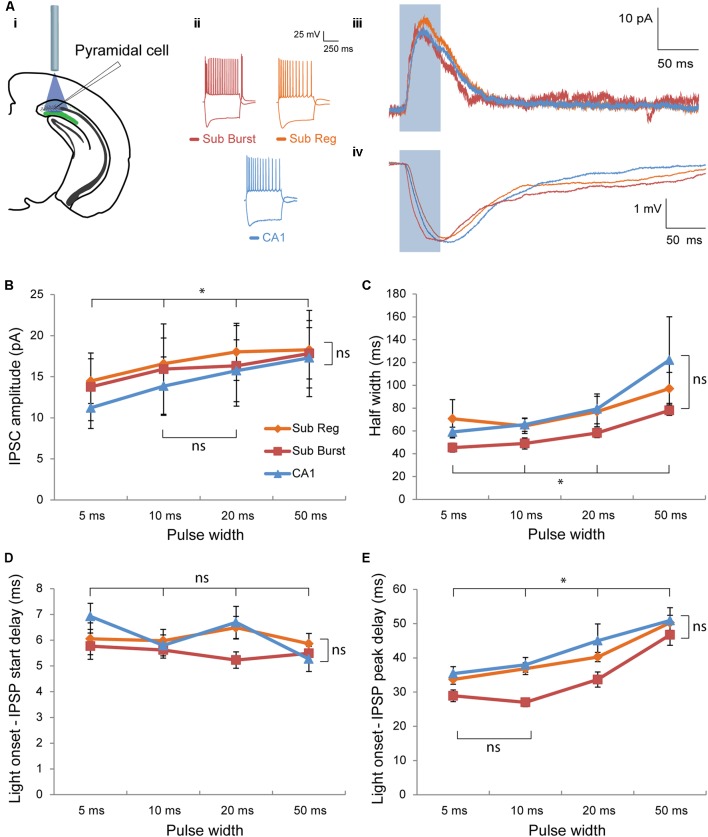
Post-synaptic responses elicited in pyramidal cells by optogenetic activation of Chrna2 cells. **(Ai–Aiv)** Representative pyramidal cells recorded in subiculum and CA1. **(Ai)** Diagram depicting optogenetic patch clamp experiment. **(Aii)** Responses to depolarizing and hyperpolarizing current steps in cc recorded in a representative subiculum burst firing (red), subiculum regular firing (orange), or CA1 (blue) pyramidal cell. Color scheme maintained throughout. **(Aiii)** Corresponding representative mean inhibitory post-synaptic current (IPSC) in response to a 50 ms light pulse (blue box) recorded in vc at an h.p. of –60 mV. **(Aiv)** Corresponding representative mean inhibitory post-synaptic potential (IPSP) in response to a 50 ms light pulse (blue box) recorded in cc at an h.p. of –60 mV. **(B)** Mean IPSC amplitude for all cells recorded across cell type and pulse width. Subiculum regular, *n* = 17; subiculum burst, *n* = 12; CA1, *n* = 10. Color legend maintained throughout the figure. **(C)** Same as in **(B)** for mean half width. **(D)** Same as in **(B)** for mean delay between light pulse onset and IPSP start. **(E)** Same as in **(B)** for mean delay between light pulse onset and IPSP peak. Error bars = SEM. ^∗^*p* < 0.05. ns, not significantly different, *p* > 0.05.

To characterize the post-synaptic responses elicited in pyramidal cells by Chrna2 cell activation, putative pyramidal cells were patched in subiculum and CA1 [*n* = 39: 29 subiculum (17 regular and 12 burst firing), 10 CA1]. The first aim of these experiments was to compare the responses elicited in CA1, subiculum regular firing or burst firing pyramidal cells. Optogenetic activation elicited inhibitory post-synaptic responses in all three cell types (**Figures 3Ai–Aiv**). The amplitude of the inhibitory post-synaptic current (IPSC) was not significantly different between cell types, but did increase significantly with increasing pulse width, with the exception of between 10 and 20 ms [**Figures 3Aiii,B**; two-way ANOVA with Bonferroni–Holm corrections. Interaction (*F*(3.4) = 1.1) and cell type main effect (*F*(2) = 0.9: *p*’s > 0.05). Pulse width main effect: *F*(1.7) = 18.6, *p* < 0.01 with *post hoc t*-test with Bonferroni correction: *t*(52)’s ≥ 2.9, *p* < 0.05 for all comparisons except 10 vs 20 ms, *t*(52) = 0.9, *p* > 0.05]. The results were similar when amplitude was measured in cc, with no difference between cell type and with amplitude increasing for every increase in pulse width [two-way ANOVA with Bonferroni–Holm corrections. Interaction (*F*(2.8) = 3.4) and cell type main effect (*F*(2) = 2.4): *p*’s > 0.05. Pulse width main effect: *F*(1.4) = 60.8, *p* < 0.01 with *post hoc t*-test with Bonferroni correction: *t*(76)’s ≥ 5.6, *p* < 0.001 for all comparisons]. To determine whether the kinetics of the response differed between cell types or pulse widths, half width was assessed. The half width of the inhibitory post-synaptic potential (IPSP) was not significantly different between cell types, but did increase significantly with increasing pulse width [**Figures 3Aiv,C**; Kruskal–Wallis test with Bonferroni–Holm corrections for interaction, *H*(3)’s ≤ 0.65 and cell type main effect, *H*(2) = 5.7: *p*’s > 0.05. Friedman test with Bonferroni–Holm correction for pulse width main effect: csqr(3) = 25.7, *p* < 0.01 with *post hoc* Wilcoxon with Bonferroni correction: *W*(39)’s ≤ 247, *p* < 0.05 for all comparisons]. Comparison of IPSP decay time yielded similar results with no significant difference between cell type, and a longer decay time for 50 ms pulse than for 5, 10, or 20 ms pulses [Kruskal–Wallis with Bonferroni–Holm corrections for interaction, (*H*(3) ≤ 4.1) and cell type main effect, (*H*(2) = 5.36): *p* > 0.05. Friedman test with Bonferroni–Holm correction for pulse width main effect: csqr(3) = 15.3, *p* < 0.01, with *post hoc* Wilcoxon with Bonferroni correction: *W*(38)’s ≤ 72, *p* < 0.05 for 50 ms vs 5, 10, and 20 ms; *W*(38)’s ≥ 208, *p* > 0.05 for remaining comparisons]. To determine if response timing differed between cell types or pulse widths, delay between light onset and IPSP start or IPSP peak was assessed (**Figures 3Aiv,D,E**). For both parameters, the delay was not significantly different between cell types [for both: two-way ANOVA with Bonferroni–Holm corrections. Interaction (delay to start: *F*(5.3) = 1.7; delay to peak F(4.7) = 1.8) and cell type main effect [*F*(2)’s ≤ 4.1: *p*’s > 0.05]. For delay to IPSP start, there was also no significant difference between pulse widths [two-way ANOVA with Bonferroni–Holm correction. Pulse width main effect: *F*(2.7) = 2.7, *p* > 0.05]. For delay to IPSP peak, the delay increased significantly with increasing pulse width, with the exception of between 5 and 10 ms [two-way ANOVA with Bonferroni–Holm correction. Pulse width main effect: *F*(3.4) = 84.6, *p* < 0.01, with *post hoc t*-test with Bonferroni correction: *t*(74)’s ≥ 4.7, *p* < 0.05 for all comparisons except 5 vs 10 ms, *t*(74) = 1.5, *p* > 0.05]. Overall, there was no significant difference in the amplitude, kinetics, or timing of the inhibitory post-synaptic responses elicited by the activation of Chrna2 cells in CA1, subiculum regular firing or subiculum burst firing pyramidal cells.

**FIGURE 4:**
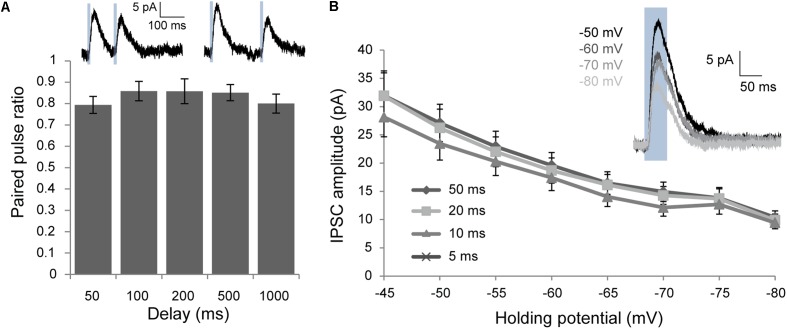
Voltage clamp characterization of post-synaptic responses elicited by optogenetic activation of Chrna2 cells. **(A)** Mean paired-pulse ratio for delays for 50–1000 ms. *n* = 9 for 50 and 100 ms, *n* = 6 for 200, 500, and 1000 ms. Error bars = SEM. ^∗^*p* < 0.05. Inset (above): Example mean IPSCs recorded at an h.p. of –60 in response to two 10 ms blue light pulses (blue boxes) with a delay of 100 ms (i) or 200 ms (ii). **(B)** Mean IPSCs recorded at h.p.’s from –45 to –80 mV for 5–50 ms light pulses. *n* = 16. Error bars = SEM. Inset: Example mean IPSC in response to a 50 ms light pulse (blue box) at h.p.’s of –50 to –80 mV.

To further characterize the inhibitory post-synaptic responses elicited by Chrna2 cell activation, paired-pulse recordings were performed over a range of delays to investigate any short-term plasticity in the response (*n* = 9 for 50 and 100 ms, *n* = 6 for 200, 500, and 1000 ms). Modest, but statistically significant, paired-pulse depression was observed for all pulse delays tested from 50 to 1000 ms [**Figure 4A**; one-sample two-tailed *t*-tests with Bonferroni–Holm correction: *t*(5 or 8) ≥-3.0, *p* < 0.05 for all]. IPSCs were also assessed over a series of h.p.’s from -45 to -80 mV to determine the reversal potential (**Figure 4B**; *n* = 16). IPSC amplitude decreased from -45 to -80 mV for all h.p.’s, but did not reached 0 or reverse.

**FIGURE 5:**
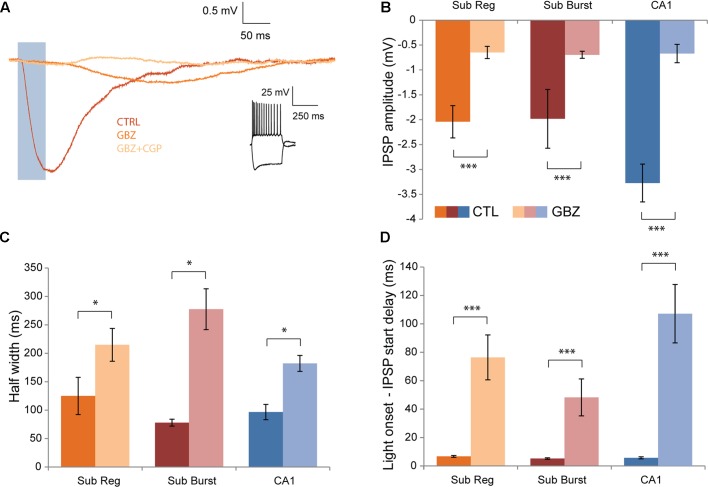
Effects of GABA_A_R and GABA_B_R antagonists. **(A)** Recordings from a representative pyramidal cell showing mean IPSP in response to a 50 ms light pulse (blue box) at an h.p. of –60 mV recorded in a control aCSF (CTRL), after the addition of gabazine (GABA_A_R antagonist) (GBZ), and after the subsequent addition of CGP-55845 (GABA_B_R antagonist)(GBZ+CGP). Inset (right): Responses to depolarizing and hyperpolarizing current steps in cc. **(B)** Mean IPSP amplitude recorded in control conditions and after gabazine application. Subiculum regular, *n* = 7; subiculum burst, *n* = 4; CA1, *n* = 3. Color legend maintained throughout the figure. **(C)** Same as in **(B)** for mean half width. **(D)** Same as in **(B)** for mean delay between light pulse onset and IPSP start. Error bars = SEM. ^∗^*p* < 0.05. ^∗∗∗^*p* < 0.001.

To determine what receptors may mediate the post-synaptic response to Chrna2 optogenetic-cell activation, responses were recorded after bath application of gabazine, a GABA_A_R antagonist, and CGP-55845, a GABA_B_R antagonist. Application of gabazine blocked a significant component of the response, leaving a smaller, slower component in the majority of cells (**Figure 5A**). The proportion of cells in which a component remained after gabazine application was greatest for the longest pulse width examined (50 ms) (cells with a response remaining after gabazine: 5 ms, *n* = 10/15; 10 ms, *n* = 11/15; 20 ms, *n* = 10/15; and 50 ms, *n* = 14/15). The smaller amplitude (mean = -0.67 ± 0.07), slower response was completely abolished by subsequent application of CGP-55845, and thus is GABA_B_R mediated. Response amplitude, kinetics and timing were assessed to examine the GABA_B_R-mediated component in further detail and to determine whether it might be different between subiculum regular and burst firing and CA1 pyramidal cells (*n* = 14: subiculum regular, *n* = 7; subiculum burst, *n* = 4; CA1, *n* = 3; **Figures 5B–D**). IPSP amplitude was significantly smaller after gabazine application compared to control conditions, but was not significantly different between cell types [**Figure 5B**; two-way ANOVA with Bonferroni–Holm corrections. Interaction and cell type main effect: *F*(2)’s ≤ 2.3, *p*’s > 0.05. Drug main effect: *F*(1) = 46.7, *p* < 0.001]. Response half width (**Figure 5C**) and decay time were significantly longer after gabazine application compared to control conditions, but were not significantly different between cell types [for both—two-way ANOVA with Bonferroni–Holm corrections. Interaction and cell type main effect: *F*(2)’s ≤ 0.5, *p*’s > 0.05. Drug main effect: *F*(1)’s ≥ 8.1, *p*’s < 0.05]. Delay from light onset to IPSP start (**Figure 5D**) and to IPSP peak were also significantly longer after gabazine application compared to control conditions, but was not significantly different between cell types [for both—two-way ANOVA. Interaction and cell type main effect: *F*(2)’s ≤ 2.3, *p*’s > 0.05. Drug main effect: *F*(1)’s ≥ 44.9, *p* < 0.001]. Overall, the GABA_B_R-mediated component of the post-synaptic response elicited by Chrna2 cell activation is similar in amplitude, kinetics and timing for CA1 and subiculum regular and burst firing pyramidal cells.

## Discussion

Chrna2 was recently discovered to be a specific genetic marker for CA1 OLM interneurons (Leão et al., 2012), interneurons which are ideally positioned to modulate direct EC input and regulate theta rhythm. The goal of this study was to investigate Chrna2 cells in the subiculum. The findings allow characterization and comparison of Chrna2 cells and their input to pyramidal cells in the subiculum and CA1, and have implications for the understanding of the role of Chrna2 cells in subicular function.

### Characterization of Subiculum Chrna2 Cells

Chrna2 cells in the subiculum were characterized according to their Som expression and electrophysiological and morphological properties, and these properties were compared to those of Chrna2 cells in CA1. The majority of subiculum Chrna2 cells expressed Som, as did those in CA1. This finding is consistent with the characteristic Som expression associated with OLM interneurons in CA1 and CA3 (Morrison et al., 1982; Somogyi et al., 1984; Naus et al., 1988; Katona et al., 1999; Losonczy et al., 2002; Klausberger et al., 2003; Leão et al., 2012). In subiculum, a larger proportion of the Som-expressing neurons also expressed Chrna2 as compared to CA1. This proportionally larger number of Chrna2 cells in the subiculum than CA1 may indicate a lower diversity of populations of Som cells in the subiculum.

The electrophysiological properties of Chrna2 cells in the subiculum and CA1 were homogeneous and not significantly different. Chrna2 cells in both sub-regions displayed characteristic features of CA1 OLM interneurons: slow, spontaneous firing at *V*_r_, regular spiking with some frequency accommodation in response to depolarizing current steps, and a sag in their responses to hyperpolarizing current steps (McBain et al., 1994; Sik et al., 1995; Maccaferri and McBain, 1996; Maccaferri et al., 2000; Gloveli et al., 2005; Leão et al., 2012; Chittajallu et al., 2013). The sag is associated with *I*_h_ current, a hyperpolarization-activated conductance believed to play a role in the theta frequency firing recorded in OLM interneurons (Gillies et al., 2002; but see Maccaferri and McBain, 1996; Kispersky et al., 2012).

Finally, the morphology of Chrna2 cells in subiculum and CA1 was characterized and compared. CA1 Chrna2 cells displayed a morphology that was similar to that described in previous studies of CA1 OLM interneurons (Cajal, 1911; Lorente de Nò, 1934; McBain et al., 1994; Sik et al., 1995; Maccaferri et al., 2000; Losonczy et al., 2002; Leão et al., 2012). Subiculum Chrna2 cells displayed some similarities to as well as some significant differences from those in CA1. Their somas and dendrites were similarly located in their respective deep layer and showed no difference in the complexity of their dendritic branching, however, they occupied a significantly broader area than in CA1. This difference may be reflective of the area occupied by pyramidal cell axon collaterals in each sub-region. Recent studies investigating the morphology of Chrna2-Martinotti cells in the somatosensory cortex (Wang et al., 2004) and the auditory cortex (Hilscher Markus et al., 2017) have shown that the dendrites of these cells have a columnar organization, suggesting that while they modulate inputs from several layers, it is restricted within the cortical column. In line with these observations and previous findings, we found that in CA1, pyramidal cell axon collaterals are largely restricted to stratum oriens (Knowles and Schwartzkroin, 1981; Tamamaki et al., 1987). These axon collaterals have been shown to be the primary source of excitatory inputs to CA1 OLM interneuron dendrites (Lacaille et al., 1987; Blasco-Ibáñez and Freund, 1995; Maccaferri and McBain, 1995; Ali and Thomson, 1998; Sun et al., 2014), which are similarly restricted horizontally in stratum oriens (McBain et al., 1994; Sik et al., 1995; Leão et al., 2012). In subiculum, pyramidal cell axon collaterals occupy a wider area, covering multiple layers (Harris et al., 2001). This may be associated with the broader area occupied by Chrna2 cell dendrites found in this study. A similar arrangement has been described in CA3 where the broader dendritic tree of OLM interneurons is mirrored by the broader expanse of CA3 pyramidal cell axon collaterals (Gulyás et al., 1993; Müller and Remy, 2014). It is important to note, however, that the inputs to Chrna2 cells have yet to be specifically studied in subiculum, so this hypothesis is made on the assumption that they also receive substantial input from pyramidal cell axon collaterals. This assumption could be investigated in future work. Notably, the axons of subiculum Chrna2 cells also displayed more proximal axonal branching than those in CA1, with more extensive projections within the molecular layer, as well as more variable superficial projections. Thus, whereas CA1 Chrna2 cells appear to predominantly target the distal dendrites of pyramidal cell dendrites, subiculum Chrna2 cells may also target the proximal dendrites. They may also target other interneurons in the molecular layer. Overall, subiculum Chrna2 cells may provide a more widespread modulatory influence on the excitatory input to the pyramidal cells of this area. As these differences in axonal morphology may result in differences in the inhibitory influence of Chrna2 cells on pyramidal cells in CA1 and subiculum, pyramidal post-synaptic responses were characterized.

### Chrna2 Cell-Mediated Post-synaptic Responses

An optogenetic technique was used to activate Chrna2 cells and post-synaptic responses were recorded in CA1 pyramidal cells as well as from subiculum regular and burst firing pyramidal cells. Responses in both regions were inhibitory, as expected from a previous study in CA1 (Leão et al., 2012) and the delay between light pulse onset and response start was consistent with a post-synaptic response (Amilhon et al., 2015). Generally, longer pulse widths produced larger amplitude responses with longer half widths, decay times, and delays between light pulse onset and response peak, consistent with longer pulse widths eliciting more spikes in Chrna2 cells. The amplitude, kinetics, and timing of the response were not significantly different between CA1, subiculum regular firing, and subiculum burst firing pyramidal cells. As IPSC size and kinetics measured in the soma are believed to be associated with an interneuron’s target region on the dendritic tree (Maccaferri et al., 2000), the similarity in the post-synaptic responses in subiculum and CA1 suggests that Chrna2 cells inhibit the same segments of the pyramidal cell dendritic tree. Thus, the observed morphological differences in the axonal arborizations of Chrna2 cells in each region may not reflect differences in how Chrna2 cells target pyramidal cells. It may instead reflect a more extensive role for subiculum Chrna2 cells in modulating local interneurons in the molecular layer and other excitatory input (i.e., lateral and medial EC), a hypothesis which could be investigated in future studies.

Paired-pulse recordings revealed modest paired-pulse depression, consistent with previous work in CA1 (Maccaferri et al., 2000; Minneci et al., 2007). This suggests that Chrna2 cells may lose some of their modulatory effect during repetitive stimulation, such as during theta rhythm. However, this minor depression may be counterbalance by the facilitation seen in the excitatory input from CA1 pyramidal cells to OLM interneurons over repeated stimulation (Ali and Thomson, 1998; Losonczy et al., 2002; Kim, 2014).

Post-synaptic responses were also recorded at h.p.’s from -45 to -80 mV to determine their reversal potential. The inhibitory response decreased as the h.p. was more negative, but did not reverse. This was surprising as the range of h.p.’s examined were close to the reversal potential for chloride, the ion conductance associated with GABA_A_Rs (Olsen and DeLorey, 1999). This may have been the result of inefficient recording of GABA_A_R conductances generated in distal dendrites due to poor vc at these distal sites The response may also involve GABA_B_Rs, which have a potassium-mediated reversal potential at closer to -90 mV (Sodickson and Bean, 1996; Olsen and DeLorey, 1999; Degro et al., 2015). In the majority of cells, the GABA_A_R antagonist gabazine attenuated a significant component of the response, but a smaller amplitude, slower component, consistent with a GABA_B_R-mediated response, remained (Sodickson and Bean, 1996; Booker et al., 2013; Degro et al., 2015). This remaining component was reversibly abolished by the addition of the GABA_B_R antagonist CGP-55845. This study is the first to show a GABA_B_R component in OLM interneuron post-synaptic inhibition. This GABA_B_R-mediated response was significantly smaller in amplitude and displayed slower kinetics and longer delays to response start and peak. No significant differences were found in amplitude, kinetics, or timing between cell types, confirming that Chrna2 cells elicit similar responses in both CA1 and subiculum and both subiculum pyramidal cell types. Overall, these results suggest that Chrna2 cell-mediated post-synaptic responses in both subiculum and CA1 are composed of both fast GABA_A_R and slow GABA_B_R mediated components.

### Implications for the Role of Subiculum Chrna2 Cells

Apart from some morphological differences, the results of this study indicate that subiculum Chrna2 cells are generally similar to those in CA1, particularly in their effects on pyramidal cells. These similarities suggest that they may also serve similar roles. Two areas of particular interest are roles in theta rhythm and memory function.

OLM interneurons in CA1 are believed to play a role in the modulation of theta oscillations. They have been found to increase their firing during theta rhythm and phase lock to the trough of the theta cycle (Klausberger et al., 2003; Varga et al., 2012; Huh et al., 2016). Rhythmic inhibition from OLM interneuron may phase-modulate EC input, which also plays a role in theta generation (Kamondi et al., 1998; Buzsáki, 2002; Mizuseki et al., 2009). Given the similarities this study has found in the firing of and inhibitory post-synaptic responses elicited by Chrna2 cells in the subiculum and CA1 subiculum Chrna2 cells may also phase-modulate EC input. Amilhon et al. (2015) found that silencing Som-expressing interneurons during the activation of EC projections significantly modulated the effect of EC input on theta rhythm in CA1/subiculum. Future work focusing specifically on Chrna2 cells could determine what role this sub-population of Som-expressing interneurons plays in this effect.

OLM interneurons in CA1 are also believed to play a role in memory. Lovett-Barron et al. (2014) found that inhibition of direct EC input to the distal tuft of CA1 pyramidal cells was necessary for the formation of contextual fear memories, inhibition they suggest is provided by OLM interneurons. Furthermore, Som has also been shown to be necessary for the acquisition of contextual fear memories (Kluge et al., 2008). Given the similarity in their post-synaptic responses and Som expression, subiculum Chrna2 cells could also contribute to memory formation by excluding the discrete sensory information provided by direct EC input or through Som-mediated modulation, particularly since the subiculum is also known to play a role in spatial learning and memory (Galani et al., 1997; Laxmi et al., 1999; Oswald and Good, 2000; O’Mara et al., 2009). A loss of CA1 OLM interneurons has also been associated with cognitive decline (Stanley et al., 2012). A similar change in Chrna2 cells in subiculum may also give rise to cognitive impairments. This idea is supported by studies which find that the subiculum is among the earliest brain regions affected in Alzheimer’s disease (Adachi et al., 2003; de la Prida et al., 2006; Scher et al., 2011; Goutagny et al., 2013; Trujillo-Estrada et al., 2014), and that Som-expressing interneurons in subiculum are particularly vulnerable (Trujillo-Estrada et al., 2014).

Overall, this study has provided the first in-depth characterization of Chrna2 cells in the subiculum and has enhanced our understanding of how this interneuron population may contribute to subiculum function. The findings suggest that subiculum and CA1 Chrna2 cells are largely equivalent, modulate pyramidal cell activity through GABA_A_Rs and GABA_B_Rs, and are likely to play similar roles both sub-regions, roles which may include the regulation of theta rhythm and a contribution to learning and memory functions.

## Author Contributions

HN was the primary contributor to project design, data acquisition, analysis and interpretation, and manuscript preparation. SW was the primary contributor to project conception and significantly contributed to project design, data interpretation, and critical revision. BA, FM, and SB contributed significantly to data acquisition, analysis and interpretation, and critical revision.

## Conflict of Interest Statement

The authors declare that the research was conducted in the absence of any commercial or financial relationships that could be construed as a potential conflict of interest.

## Footnotes

1 http://imagej.nih.gov/ij/

2 http://vassarstats.net/
